# A comparative study on the PSE-like chicken breast meat protein isolate-sunflower oil emulsion stability, rheological properties and gel properties by different additions of l-Lysine

**DOI:** 10.1016/j.fochx.2025.102684

**Published:** 2025-06-21

**Authors:** Lin-Meng Wang, Yi-Xue Zhang, Qing Yang, Ke Li, Wu-Chao Ma, Yan-Hong Bai

**Affiliations:** College of Food and Bioengineering, Key Laboratory of Cold Chain Food Processing and Safety Control, Ministry of Education, Zhengzhou University of Light Industry, Ke Xue Road No. 136, Zhengzhou 450001, PR China

**Keywords:** l-lysine, PPI, Sunflower oil, Emulsion stability, Emulsion gel

## Abstract

In order to improve the emulsion gel properties of PSE (pale, soft, and exudative)-like chicken meat protein isolate (PPI), effect of different addition of l-lysine (L-Lys) on the emulsifying and gel properties of PPI-sunflower oil emulsion were studied by comparing control groups (pure PPI groups (PPI), only added L-Lys (Lys) and sunflower oil (SO)). Adding L-Lys to the PPI emulsion resulted in a decrease in particle size and uniform distribution, the stability of emulsion was increased, and the gel exhibited higher elastic (G′), better stability and more compact microstructure was formed. Especially when 0.2 % L-Lys was added to the emulsion system, the particle size of emulsion was reduced by 76.16 %, the emulsion gel strength was increased by 286.84 %, the centrifugal loss was reduced by 51.98 % (*P* < 0.05) compared with PPI. Therefore, L-Lys can be viewed as a viable method for enhancing the functional properties of PPI.

## Highlights

• The PPI emulsion with 0.2 % L-Lys added had better physical stability.
• The addition of L-Lys promoted PPI adsorption at the oil-water interface.
• L-Lys enhanced the network structure of PPI emulsion gels.
• The addition of 0.2 % L-Lys reduced centrifugal loss of PPI emulsion gel by 51.98 %.

## Graphical abstract

**Figure:**
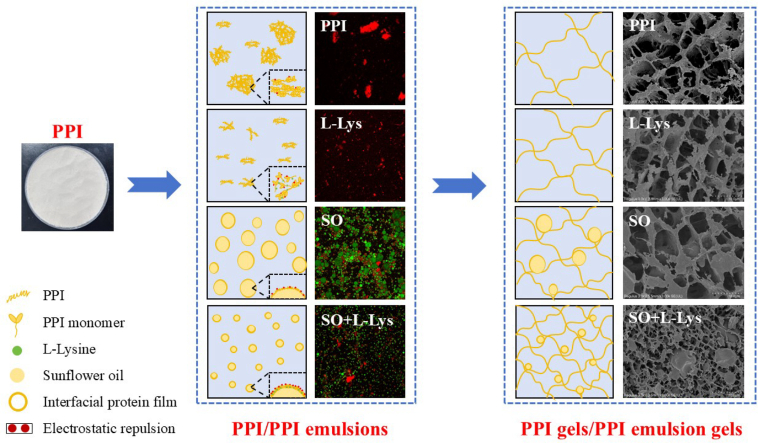

## 1 Introduction

Pale, soft, and exudative (PSE)-like chicken meat is a heterogeneous meat with a rapid decrease in muscle pH due to pre-slaughter stress and post-slaughter high-temperature conditions, this leads to protein denaturation, impairing functional properties such as solubility, gelation, and emulsification (Gao et al., 2023; Lu et al., 2022; Yang et al., 2021). The incidence of PSE-like meat is high, with over 40 % of chicken exhibiting PSE-like characteristics during the summer, resulting in annual economic losses of approximately $200 million to the meat industry (Dong et al., 2020; Lee & Choi, 2021; Li, Li, et al., 2022). As the growth of the population, the demand of meat production, particularly high-quality protein also will continue to grow (Su et al., 2024). In addition, the impairment of functional properties of meat proteins will also cause the gap of animal-derived protein to continue to expand. PSE-like meat is regarded as a high-quality source of animal protein because of its high protein content (20 %–23 %), low price, no religious and cultural restrictions, and favorable digestive properties (Li, Wang, et al., 2023). PSE-like chicken meat protein has a wide range of potential applications in the food industry, and can be used to prepare bioactive packaging materials, increase the added value of products, and promote the reuse and sustainable development of protein resources, which will bring significant economic and social benefits to the food industry (Li, Fan, et al., 2022; Zhao et al., 2020; Zhu et al., 2025). Therefore, it is very important to explore ways to enhance its functional properties, improve the processing efficiency and reduce waste and loss of animal-derived protein.

Our previous studies focused on improving the gel properties of PPI obtained by ultrasound-assisted alkaline extraction (UAE). We found that the addition of basic amino acids, especially L-lysine (L-Lys), significantly enhanced the gel properties of PPI (Li, Wang, et al., 2023). Additionally, the gelation properties of PPI were enhanced by adding various vegetable oils (peanut oil, corn oil, soybean oil and sunflower oil), especially sunflower oil. This study also found that emulsion stability had a significant effect on the gel properties of emulsion gels (Li, Wang, et al., 2024). Therefore, constructing a PPI emulsion with good stability is crucial for developing the emulsion gel system and enhancing PPI gel properties. Relevant studies have shown that basic amino acids increase the pH of protein solution due to their charge characteristics and functional groups, which enhances the adsorption capacity of proteins at the oil-water interface, helping to form a more stable interfacial membrane. It also promotes electrostatic repulsion between emulsion droplets, reduces interfacial tension between oil and water, and inhibits droplet aggregation and emulsion flocculation (Gao et al., 2025). Which has been widely used to improve the stability of animal and plant proteins prepared emulsions and develop emulsified meat products (Zhu et al., 2024).

L-Lys is an industrially producible and inexpensive amino acid that enhances the physicochemical properties of muscle proteins, including solubility, gel, emulsion and texture properties (Zhang et al., 2020). It also improves the color and tenderness of meat products and inhibit the oxidation of protein and fat (Bao et al., 2021; Zhang, Zhang, et al., 2021; Zhu et al., 2018). Which has a broad application prospect in the food industry. Combined our previous studies, L-Lys has a superior proteolytic ability compared to other basic amino acids, which helps to promote the formation of interfacial films (Li, Wang, et al., 2023). In addition, L-Lys contains both hydrophilic and hydrophobic groups, which are similar to surfactants, and theoretically possesses a superior ability to stabilize oil-water emulsions (Zhu et al., 2019). It has been reported that L-Lys significantly improves the emulsion stability of myosin and myofibrillar protein (MP), as well as the texture and gel properties of emulsified sausage (Guo et al., 2021; Hu et al., 2022; Li, Fan, et al., 2023; Zhang, Xu, et al., 2021). These findings demonstrated the very important practical value of L-Lys in the field of emulsion stability of MP and emulsified meat products. Currently, the studies on the properties of PPI emulsions through L-Lys addition are still limited. In the existing research field, the majority of studies tend to focus on a single dimension, exploring the stabilization mechanisms of protein emulsions solely from the perspective of emulsion stability or studying the stabilization mechanism of emulsion gels by focusing only on the properties of protein gels. Few studies have managed to break through this limitation and provide a comprehensive and in-depth analysis of the properties of thermally induced emulsion gels from the unique perspecti*v*e of emulsion stability (Han, Li, et al., 2021; Zhu et al., 2022). In addition, the comparative analyses between the properties of protein gels and the properties of protein emulsion gels need to be further studied. This is particularly important for de*v*eloping the theory of protein emulsion gels by further exploring the mechanism of the intrinsic relationship between emulsion stability and heat-induced emulsion gel formation based on PPI constructs.

Therefore, in this study, the protein solution-emulsion-gel/emulsion gel system was constructed with pure PPI, only L-Lys (0.05 %, *w*/*v*) added and only sunflower oil (20 %, *w*/w) added PPI as controls. The emulsion stability, emulsion gel properties and structural properties were evaluated. To elucidate the effects of L-Lys (0.05 %, 0.1 %, 0.15 %, and 0.2 %, *w*/*v*) addition on the stability and gel properties of PPI-sunflower oil (20 %, *w*/w) emulsions. The purpose was to improve the processing and utilization of PSE-like chicken meat, and to provide a theoretical basis for further exploring the application of emulsion gel as a carrier for delivering bioactive substances and replacing fat.

## 2 Materials and methods

### 2.1 Materials

PSE-like chicken breast meat was selected as described in a previous study (Li, Li, et al., 2022). L-lysine and sodium dodecyl sulphate (SDS) were supplied by Yuanye Biotechnology Co., Ltd. (Shanghai, China). The stains Nile red and Nile blue were supplied by Aladdin Biochemistry and Technology Co., Ltd. (Shanghai, China). Sunflower oil was purchased at a local supermarket (Zhengzhou, China). All reagents were of analytical grade.

### 2.2 Extraction of PPI

The PPI was prepared via UAE, as depicted in Fig. S1 of the Supplementary Material, with the entire process conducted at 4 °C. The resultant PPI powder was stored in self-sealing bags for subsequent analysis (Zou et al., 2018).

### 2.3 Preparation of emulsion and emulsion gel

As shown in Supplementary Material Fig. S1, the PPI solution was prepared using a phosphate buffer solution (20 mmol/L Na_2_HPO_4_/NaH_2_PO_4_, 0.6 mol/L NaCl, pH 7.0), sunflower oil (20 %, *w*/w) and different ratios of L-Lys (0.05 %, 0.1 %, 0.15 %, and 0.2 %, *w*/*v*). The entire experimental process is completed at a constant temperature of 4 °C. The emulsion was then prepared by homogenizing (10,000 r/min, 2 min) using a homogenizer (Ultraturrax T25, IKA, Staufen, Germany). The specific ratios of PPI, L-Lys, and sunflower oil are presented in Table 1.

**Table 1:** Ratio of sunflower oil and L-Lys added to samples from different treatment groups.

Sample	PPI/mg·mL^−1^	Sunflower oil /%	L-Lys/%
PPI	50	/	/
Lys	50	/	0.05
SO	50	20	/
0.05–20	50	20	0.05
0.1–20	50	20	0.1
0.15–20	50	20	0.15
0.2–20	50	20	0.2

The PPI solution and PPI emulsion were centrifuged for 3 min (200 ×*g*) to remove air bubbles. The samples were then heated from 25 °C to 80 °C and held at 80 °C for 30 min to form gels, followed by cooling and storage at 4 °C overnight. The PPI gels were transferred to room temperature for 30 min before measuring the indexes for subsequent analysis (Zhao et al., 2017).

### 2.4 Emulsion emulsifying activity index (EAI) and emulsifying stability index (ESI)

The absorbance of the sample (50 μL) mixed with 0.1 % SDS (5 mL) was measured at 500 nm using a UV spectrophotometer (TU-1810, General Instrument, China), and the EAI and ESI were subsequently calculated (Li, Zhao, et al., 2024).

### 2.5 Emulsion turbiscan stability index (TSI)

A 20 mL sample of fresh emulsion was scanned using a Turbiscan Lab Expert analyzer (Formulaction, Toulouse, France). The total scanning time was 1 h (25 °C) to obtain the TSI value (Li, Li, et al., 2020).

### 2.6 Emulsion particle size and zeta potential

The emulsion concentration was adjusted to 10 mg/mL, and both the particle size and particle size distribution were determined using a laser particle sizer (LS 13320/ULM2, Beckman Coulter, USA).

The zeta potential of the emulsion (1 mg/mL) was determined using a Zetasizer (Nano-ZS90, Malvern Instruments, UK) (Li, Zhao, et al., 2024).

### 2.7 Emulsion microstructure

The emulsion samples (approximately 20 μL) diluted to 10 mg/mL were placed in the center of microscope slides. The microstructure of these emulsions was examined using an optical microscope (PH50, Phenix, China) at 100× magnification (Li, Fu, et al., 2020).

The distribution of the emulsions (10 mg/mL) was analyzed using a confocal laser scanning microscopy (CLSM) (FV3000, Olympus, Japan) equipped with a 20× objective lens. Nile red (1 mg/mL in ethanol) was used to stain the oil phase and Nile blue (1 mg/mL in distilled water) was used to stain the protein phase, and images were collected using 488 nm and 633 nm excitation wavelengths, respectively (He et al., 2024).

### 2.8 Rheological properties

As described by Li, Wang, et al., 2024 with some modifications. The apparent viscosity of the samples was measured over the range of shear frequencies from 0.1 to 1000 s^−1^ at a constant temperature of 25 °C to evaluate the effect of shear rate on the apparent viscosity of emulsions. The storage modulus (G′) and loss modulus (G′′) were determined as functions of temperature (25–80 °C) at a constant frequency of 0.1 Hz and a strain of 0.5 %. The heating rate was 2 °C/min.

### 2.9 Centrifugal loss (CL)

Approximately 5 g of the gel sample was centrifuged at 4 °C (10,000 ×g, 15 min) to remove the water. The excess water exuded from the surface was absorbed using special qualitative filter paper. The CL of the gel was calculated as the percentage ratio of the post-centrifugation gel mass to the pre-centrifugation gel mass (Zhang, Meng, et al., 2023).

### 2.10 Gel strength

The gel strength was determined using a texture analyzer (TA-XT Plus, Stable Micro Systems, UK) equipped with a P/0.5 probe, following our previously described method (Li, Wang, et al., 2024). The downward pressure distance of 20 mm and a trigger force of 5 g, the test speed was 1 mm/s, and both the pre- and post-speeds were 2 mm/s.

### 2.11 Gel microstructure

The microstructure of the gels was examined using a cryo-scanning electron microscope (Regulus 8100, Hitachi Ltd., Tokyo, Japan). The samples were initially frozen with liquid nitrogen and subsequently sublimated at −75 °C for 15 min within the sample preparation chamber. Imaging was performed at an accelerating voltage of 3 kV (Nie et al., 2024).

### 2.12 Low-field nuclear magnetic resonance (LF-NMR)

The water distribution and proportion within the PPI gel samples were measured using a LF-NMR analyzer (Niumag Electric Co., Shanghai, China) as reported by Zhang et al. (2022). The analyzer operated at a constant temperature of 32 °C. Placed a small glass vial containing 1.0 g of emulsion into an NMR tube with a diameter of 25 mm, the resonance frequency was set to 21 MHz. Other key pulse parameters are as follows: 90° pulse time = 7 μs, 180° pulse time = 14 μs, repetition time = 6000 ms, echo count = 5000, echo time = 1 ms, repeat scan times = 4 times. And used the Carr-Purcell-Meiboom-Gill (CPMG) sequence for determination.

### 2.13 Magnetic resonance imaging (MRI)

The PPI gel samples were placed at the bottom of the NMR tube as described by Shi et al. (2021). Pseudo-color images were acquired from proton density images and water distribution.

### 2.14 Fourier transform infrared spectroscopy (FTIR)

The secondary structure of PPI gel samples was determined as described by Lin et al. (2024). Freeze-dried gels (5 mg) were mixed with 500 mg KBr and finely ground, the post-press samples were scanned using a Fourier transform infrared spectrophotometer (Vertex 70, Bruker, Germany). They were obtained in the wavenumber range of 400–4000 cm^−1^ using 64 scans with a resolution of 4 cm^−1^. Subsequently, the spectral data were analyzed and fitted using PeakFit 4.12 software.

### 2.15 Statistical analysis

All experiments were performed at least in triplicate. Experimental results were expressed as the mean ± standard deviation (SD). Analysis of variance (ANOVA) was conducted using IBM SPSS Statistics 21.0, and statistical significance was set at *P* < 0.05.

## 3 Results and discussion

### 3.1 EAI and ESI

**Fig. 1:**
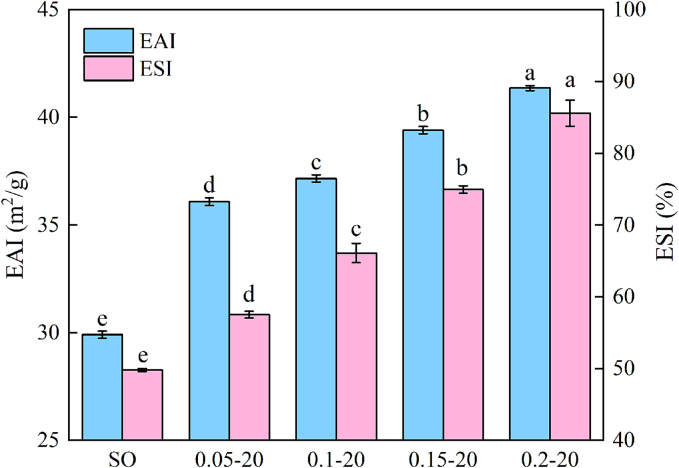
EAI and ESI of PPI emulsions in different treatment groups. Different letters (a–e) indicate significant differences between different treatment groups under the same indicator (*P* < 0.05).

EAI represents the adsorption capacity of proteins at the oil-water interface, while ESI generally characterizes the rate of phase separation between the water and oil phases during emulsion storage (Pan et al., 2024). As shown in Fig. 1, the PPI emulsion with L-Lys addition exhibited significantly higher EAI and ESI values compared to the SO group. Especially for the PPI emulsion with 0.2 % L-Lys addition, the EAI of the PPI emulsion was increased from the initial 29.90 m^2^/g to 41.36 m^2^/g (an increase of about 38.33 %), and the ESI was increased from the initial 49.78 % to 85.58 % (an increase of about 71.92 %) (*P* < 0.05). The elevated EAI and ESI indicated that L-Lys enhanced the interaction between PPI and oil and the emulsifying ability of PPI. This may be related to the combination of protein amounts at the interface, protein conformation and their interactions (Han, Xu, et al., 2021). L-Lys improves the solubility and affinity water balance of PPI, which promotes faster and more complete coverage of proteins at the O/W interface and improves emulsion stability (Guo et al., 2021; Li et al., 2018). L-Lys molecules can adsorb competitively with proteins at the oil/water interface, contributing to the formation of stronger interfacial films in emulsion droplets (Huang et al., 2021; Zhu et al., 2019). In addition, L-Lys is able to bind negatively charged residues of myosin via electrostatic interactions, thereby disrupting intramolecular and intermolecular ionic bonding and leading to the exposure of hydrophobic groups, which facilitates protein-oil interactions and promotes protein adsorption according to the research of Guo et al. (2015).

### 3.2 TSI

**Fig. 2:**
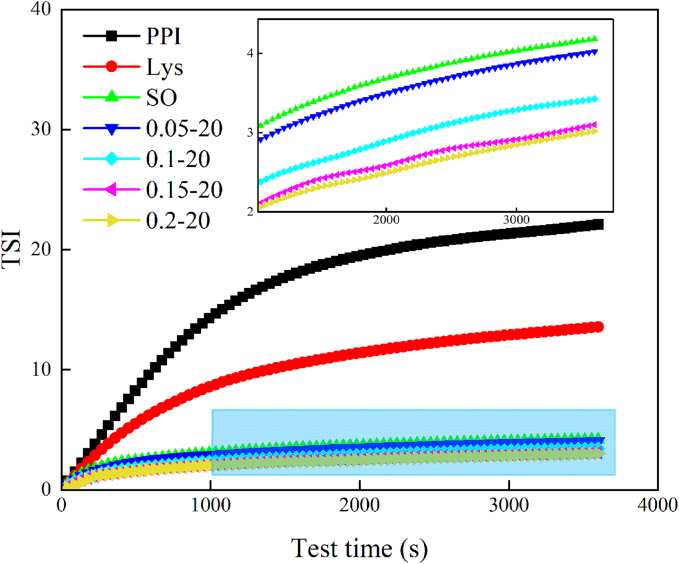
TSI of PPI solution or emulsions in different treatment groups.

The TSI value utilizes multiple light scattering to characterize particle migration and interactions and can be used to estimate emulsion stability (Zhang, Bi, et al., 2024). As shown in Fig. 2, in the initial stage of the measurement, all samples experienced a rapid instability phase. And the TSI values all increased rapidly with the increase of the measurement time, and the rate started to decelerate at 1000 s and eventually began to stabilize at 3000 s. The overall TSI values were reduced with the addition of L-Lys and sunflower oil compared to the PPI group. The order of their TSI values was PPI > Lys > SO >0.05–20 > 0.1–20 > 0.15–20 > 0.2–20, which means that the addition of L-Lys and sunflower oil is conducive to the stability of PPI. Fatty acid types in sunflower oil alter protein adsorption and conformation, thus affecting emulsion stability (Tao, Cai, Wang, Chen, Zhou, Zhang, & Xu, 2024). The structure and the makeup of the protein membrane contributed to emulsification stability (Xu et al., 2020). The addition of L-Lys after the construction of emulsion system has a more significant stabilizing effect on PPI emulsion, The TSI value of the emulsion is the lowest when the L-Lys addition is 0.2 %, which means the stability is the highest. This means that L-Lys can promote the emulsification ability of PPI emulsion. The increase in EAI value also confirms this result.

### 3.3 Emulsion particle size and size distribution

**Fig. 3:**
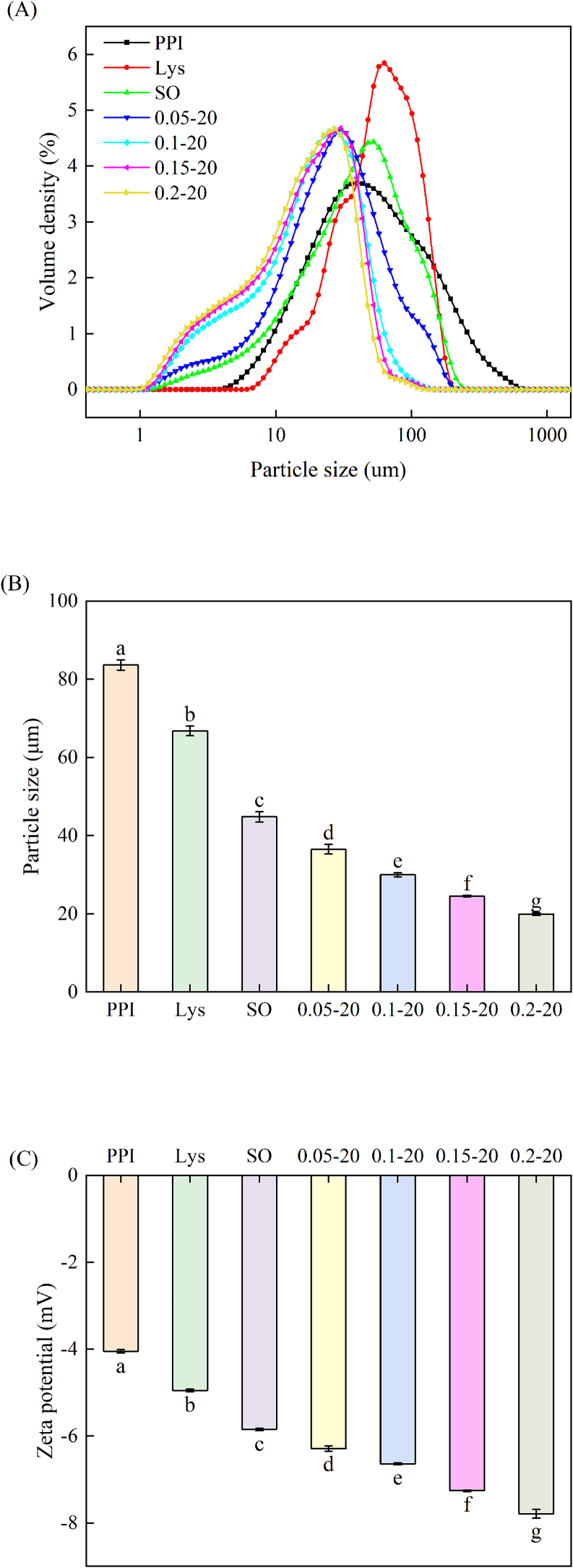
Particle size distribution (A), particle size (B) and zeta potential (C) of PPI solution or emulsions in different treatment ways. Different letters (a–g) indicate significant differences between different treatment groups under the same indicator (*P* < 0.05).

The droplet size is related to both emulsion stability and the physical properties of the emulsion-filled gel (Cai et al., 2022). As shown in Fig. 3 A, the pure PPI group solution showed a broadly distributed single peak and a relatively large particle size of 83.64 μm (Fig. 3B). The overall distribution of particle size within protein solution was narrower and shifted to the left with a relative decrease in particle size of 66.77 μm and 44.79 μm with the addition of L-Lys and sunflower oil, respectively. L-Lys was added after the construction of the emulsion system, the emulsion particle size distribution appeared to be more obviously left-shifted and the corresponding particle size was significantly reduced. Especially the emulsion with 0.2 % L-Lys addition, which had the largest left-shift and the smallest particle size, which was 19.94 μm. These results indicate that both L-Lys and sunflower oil can reduce the size of PPI molecules. In addition, L-Lys can effectively decrease the droplet size in PPI emulsion. Guo et al. (2021) also found that the addition of L-Lys reduced the droplet size in emulsion. Previous studies have shown that L-Lys could inhibit protein aggregation by altering electrostatic interactions through its own charge properties, as well as interfere with hydrogen bonding to promote proteolysis by causing dipole-dipole or ion-dipole interactions through specific structures (Li, Wang, et al., 2023). The protein adsorption on the interface film of the emulsion can inhibit aggregation between emulsion droplets (Li, Wang, et al., 2024). L-Lys had competitive adsorption with protein at the O/W interface or induced structural changes of interfacial protein, promotes the formation of stronger interfacial film (Gao et al., 2025), which further inhibits oil droplets aggregation. In addition, the size of the emulsion droplets, the degree of uniformity of distribution and the number of proteins involved in the formation of the emulsion are important factors influencing the EAI and ESI (Zhou, Zhang, et al., 2022). Generally, smaller droplet size and more uniform emulsion distribution led to higher emulsion stability. Therefore, it can be inferred that the improvement in the ESI of PPI emulsions after the addition of L-Lys is mainly due to the reduction in droplet size and the more uniform distribution of the emulsion.

### 3.4 Emulsion zeta potential

The electrostatic repulsion between proteins was further explored by measuring the zeta potential, which reflected the magnitude of the ability to inhibit protein aggregation (Han et al., 2022). As shown in Fig. 3C, the zeta potential values of all samples were negative, and the absolute values of the zeta potential of the samples increased significantly with the addition of L-Lys and sunflower oil, respectively (*P* < 0.05). This is a result of L-Lys and sunflower oil promoting protein dissociation, exposing more negatively charged amino acid residues (Li, Wang, et al., 2023; Li, Wang, et al., 2024). In addition, the absolute value of the zeta potential of the PPI emulsion further increased from 6.29 mV to 7.79 mV with increasing L-Lys addition after constructing the emulsion system with the addition of L-Lys, reaching a maximum value at 0.2 % L-Lys addition (*P* < 0.05). This is consistent with the findings of Hu et al. (2022), which found that L-Lys significantly enhanced the absolute zeta potential of soybean oil-myosin emulsion. This is due to the fact that the addition of L-Lys, as an basic amino acid, leads to an increase in the pH of the PPI emulsion, which deviates from the isoelectric point, and the droplets are more negatively charged (Zhu et al., 2019). Alternatively, L-Lys promotes the dissociation of PPI adsorbed on the surface of the protein membrane, and the negative surface charge increases (Guo et al., 2021). Generally, an increase in surface negative charges enhances the electrostatic repulsion between emulsion droplets. Strong electrostatic repulsion can effectively counteract the presence of hydrophobic attraction, thereby preventing coagulation and aggregation, and ultimately improving the stability of the emulsion (Li et al., 2017). This also provides a reasonable explanation for the above-mentioned reduction in emulsion particle size and improvement in emulsion stability due to the addition of L-Lys.

### 3.5 Emulsion rheological properties

#### 3.5.1 Emulsion apparent viscosity

**Fig. 4:**
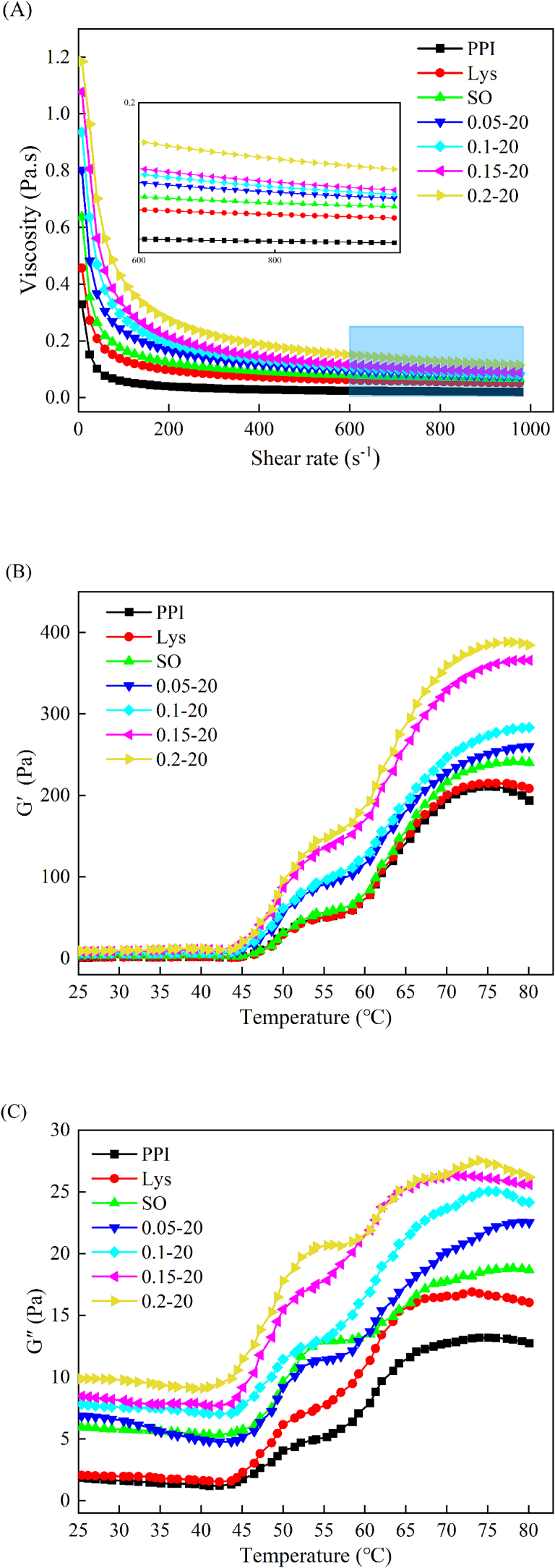
Apparent viscosity (A), storage modulus (G′) (B) and loss modulus (G″) (C) of PPI solution or emulsions in different treatment ways.

Apparent viscosity can be used to characterize the fluidity of emulsions (Pan et al., 2024). At the beginning of the shear frequency sweep, the apparent viscosity of all treatment groups was greater than that of the PPI group, and the PPI emulsion with 0.2 % L-Lys possessed the highest apparent viscosity. And as the shear frequency increased, the apparent viscosity of the protein gradually decreased, and finally stabilized after 800 s^−1^ (Fig. 4A), which is also known as shear thinning (Zhang, Bi, et al., 2024). The apparent viscosity of the treated groups remained higher than that of the control group (PPI) after plateauing, and furthermore, the synergistic treatment was more effective than the single treatment, as the overall apparent viscosity of the emulsion increased with the increase of L-Lys addition and remained the highest at 0.2 %. In general, the smaller the particle size of the emulsion, the lower the flow index and the higher the apparent viscosity (Xie et al., 2022), which concurs with the results obtained in this study with respect to the aforesaid particle size. The apparent viscosity of the PPI emulsion was further reduced by the addition of L-Lys. This phenomenon might be ascribed to the circumstance that L-Lys enhanced the adsorption capacity of PPI onto the sunflower oil droplets (increase in the EAI value). Which facilitates the production of interfacial films and the formation of the spatial site barrier, thus increasing the apparent viscosity of the emulsion (Tao, Cai, Wang, Chen, Zhou, Yang, & Xu, 2024). It may also be related to the fact that L-Lys acts as a “bridge” between two different emulsion droplets under the current conditions, thus slowing down the movement of the emulsion droplets (Zhu et al., 2019). It has additionally been observed that the correlation between the apparent viscosity and the stability of emulsion, with the higher viscosity of the emulsion being associated with better stability (Zhou, Drusch, & Hogan, 2022). Therefore, the increase in apparent viscosity for the higher L-Lys addition in this study confirms the increase in stability of the emulsion, which is in accordance with the findings obtained from TSI and ESI.

#### 3.5.2 Emulsion dynamic rheology

The rheological temperature data can also provide a reference for the formation of gel network during heat treatment (Zhang, Ni, et al., 2024). As shown in Fig. 4B, all samples showed a similar trend in G′ values. The G′ of PPI remained relatively stable at 25–45 °C. Subsequently, G ‘continued to rise to about 53 °C, and the rise of the characteristic curve was mainly due to the dimerization of myosin head and the strengthening of intermolecular cross-linking (Cao et al., 2022). When the temperature rose slowly from 54 °C to 59 °C, the myosin tail unfolded and a gel network framework initially appeared (Liu et al., 2025). And it continues to rise from 60 °C to 75 °C, which means that the cross-linking state is stronger and the system stability is enhanced (Chen et al., 2016). After 75 °C, the G′ remains in a relatively stable state. In this process, the G′ was higher than that of the PPI group with the addition of L-Lys and sunflower oil, and when the emulsion system was constructed and L-Lys was added, the G′ further increased with the increase of L-Lys addition. It has been reported that the increase in G′ values may be due to the increase in protein solution and emulsion pH caused by the addition of L-Lys, which increases electrostatic charge (Zhu et al., 2019). It is also possible that the oil droplets fill the voids within the gel matrix during thermal gelation (Zhou et al., 2017).

G′′ reflects the viscosity characteristics of the gel. As shown in Fig. 4C, the trend of the characteristic curve of G′′ is slightly different from that of G′. G″ is continuously decreasing from 25 to 45 °C and reaches the lowest value at 45 °C, and the trend is the same as that of G′ after 45 °C. It is worth noting that the treatment groups exhibited higher G″ values compared to the PPI group, and the highest G″ was found in the emulsion after adding 0.2 % L-Lys, which implies that adding L-Lys can cause more viscous behaviors.

During the entirety of the heating process, the G′ and G″ values were increased in all treatment groups and reached the maximum value at 0.2 % L-Lys addition, indicating that the addition of sunflower oil and L-Lys promoted the intermolecular interaction, which favored the cross-linking and aggregation during the heat-induced process of PPI, and the addition of L-Lys had a significant effect on the viscoelasticity and the network structure of the PPI emulsion gels. The G′ was higher than G″ for each sample, suggesting a less viscous or more elastic solid-like gel during PPI gel formation. This could be attributed to the occurrence of protein denaturation, which brings about a transformation in the emulsion system, shifting it from a relatively weak and unstructured network towards a more orderly and organized gel matrix (Lee et al., 2023). Ban et al. (2024) also showed that L-Lys increased the G′ and G″ of the emulsion gels, and suggested that this was due to the increase in pH by L-Lys and the enhancement of electrostatic repulsion within the emulsions.

### 3.6 Emulsion microstructure

**Fig. 5:**
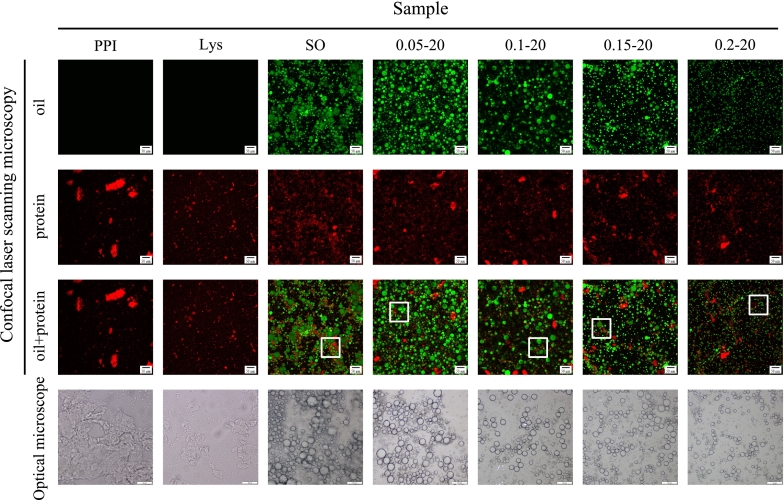
Microstructure of PPI solution or emulsions in different treatment ways.

The application of CLSM and optical microscope provides a more direct response to the characteristics of the emulsion and the distribution of the protein membrane. As shown in Fig. 5, it was observed under the optical microscope that the pure PPI solution and the PPI solution with only L-Lys added were unable to form emulsions without the addition of sunflower oil, showing chunky protein aggregates. The chunks appeared red in the CLSM images and the chunks of protein molecules in the L-Lys group were even smaller. The protein wrapped around the oil droplets (green) to form the interfacial film (red) after the addition of sunflower oil to form the emulsion, and the droplet sizes of the SO group were large and uneven in size. With the addition of L-Lys, the droplet size decreased, especially with the addition of 0.2 % L-Lys, the droplet size was minimized and uniformly distributed. The PPI formed an interfacial protein film on the oil droplet surface (the white marked part) can effectively prevent the oil droplets from aggregating in the PPI-sunflower oil emulsion, resist the deformation of oil droplets, and make the oil droplets more stable (Tao, Cai, Wang, Chen, Zhou, Yang, & Xu, 2024). With the addition of L-Lys, the distribution of interfacial protein film gradually increased, and almost every oil droplet surface was covered by a protein film in the PPI emulsion with 0.2 % L-Lys added, which was manifested by the most extensive distribution of the red area on the surface of the oil droplets. Zhu et al. (2019) reported that L-Lys has the ability to adsorb on the interface of emulsion droplets and function either in forming interfacial membrane or change the structure of interfacial proteins. Therefore, it can prove our previous conjecture that the addition of L-Lys promotes PPI adsorption at the O/W interface to form a stronger and more widely distributed interfacial protein film, which further enhances the electrostatic repulsion between the PPI emulsion droplets, leading to reduced and uniformly distributed emulsion droplets, and improves the emulsion stability. Hu et al. (2022) also found that L-Lys was effective in reducing emulsion droplet size through CLSM images. This is consistent with the above results for particle size, zeta potential and emulsion stability in this study.

### 3.7 CL

**Fig. 6:**
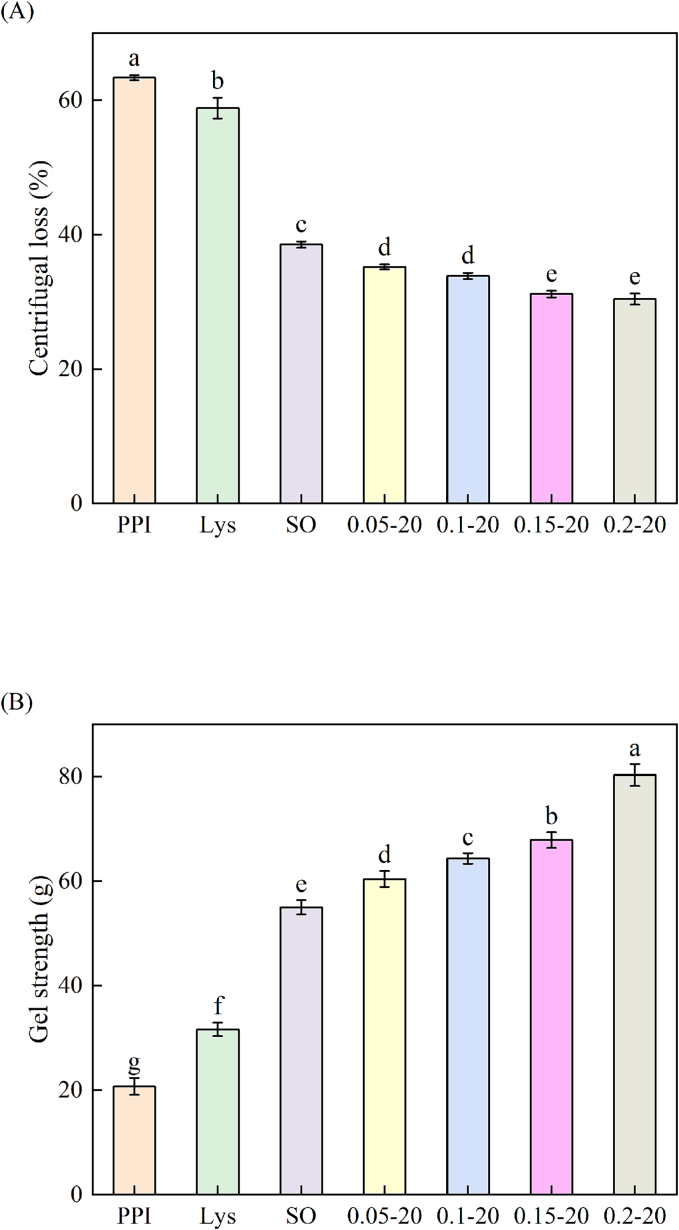
Emulsion stability (A) and gel strength (B) of PPI solution or emulsion gels in different treatment way.

Water-holding capacity (WHC) represents the capacity of proteins to retain water and is a crucial attribute for assessing gel quality, which is directly related to texture properties (Ge et al., 2023). Fig. 6A shows the CL of PPI emulsion gels after different treatments. Compared to PPI group (63.35 %), the addition of either L-Lys or sunflower oil alone helped mitigate the depletion of water content in PPI gels (58.80 % and 38.52 %, respectively), with sunflower oil having a greater effect than the addition of L-Lys (*P* < 0.05). The addition of L-Lys to the emulsion system further decreased the CL of the PPI emulsion gels, and the CL of the PPI emulsion gel reached the lowest (30.42 %) at 0.2 % L-Lys addition (*P* > 0.05). This implies that the three-dimensional network gels formed by the addition of L-Lys and sunflower oil are relatively effective in water retention, while L-Lys also contributes to the enhancement of water retention in the gel network structure of PPI emulsion and improves their stability against centrifugal deformation. Gao et al. (2023) reported that the addition of L-Lys reduced the CL of PSE-like chicken breast meat emulsion sausage. Relevant studies have shown that the WHC of protein gels is closely related to the stability of the network structure, the more stable the gel structure, the weaker the water mobility and the stronger the WHC (Cao et al., 2022). Therefore, it was speculated that the addition of L-Lys could facilitate the formation of a more compact PPI emulsion gel network and strengthen its structural stability, thus reducing water migration and improving water retention.

### 3.8 Gel strength

As shown in Fig. 6B, the gel strength of PPI was increased by the addition of both L-Lys and sunflower oil (31.64 g and 54.99 g, respectively) when compared to the gel of the untreated PPI group (20.75 g) (*P* < 0.05). And further increased after adding L-Lys to the emulsion system, especially after the addition of 0.2 % of L-Lys, L-Lys reached the maximum gel strength (80.27 g), the gel strength was improved by 45.97 % compared to the SO group with only sunflower oil, and by 286.84 % compared to the PPI group (*P* < 0.05). Both L-Lys and sunflower oil improved the gel strength of PPI gels, and sunflower oil improved better than L-Lys, and L-Lys improved the gel strength of PPI-sunflower oil emulsion gels, and the emulsion gel strength was directly proportional to the stability of the emulsion. The above rheological characterization revealed that the addition of L-Lys and sunflower oil could enhance the cross-linking of proteins in the heating process, thereby promoting formed gel/emulsion gels. It has also been reported that L-Lys and sunflower oil could promote the formation of dense gel network structure of PPI, thus improving the gel strength of PPI (Hu et al., 2022; Li, Wang, et al., 2023; Li, Wang, et al., 2024; Man et al., 2024). In addition, it was reported that small oil droplets can act as “fillers” in the voids of the gel matrix. At the same fat concentration, small oil droplets are more likely to form a firmer gel than large oil droplets (Wu et al., 2011). Which finding will be further discussed in Section 3.9.

### 3.9 Gel microstructure

**Fig. 7:**
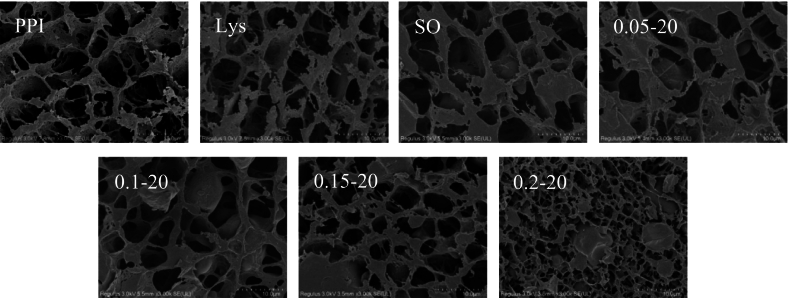
Microstructure of PPI solution or emulsion gels in different treatment ways.

As shown in Fig. 7, in the PPI group without treatment, large pores were distributed and had a relatively loose structure, the addition of L-Lys resulted in a relative decrease in the pores of the PPI gel, while the addition of sunflower oil resulted in a PPI gel with a smooth surface and larger-sized oil droplets distributed within the gel, indicating the formation of emulsion particle-filled gels. After adding L-Lys to the emulsion system, the gel network structure became denser, the pores were further reduced and uniformly distributed, and the size of the filled oil droplets inside the gel was further reduced. Especially for the PPI emulsion gel with 0.2 % of L-Lys added, which had the most homogeneous and compact gel structure, and the smallest and most uniformly filled oil droplet size. The formation of a more regular and dense spatial arrangement of proteins during thermal induction can increase the gel strength, which is consistent with the gel strength measurement result. The dense network structure can retain more water, thus reducing gel water loss (Pan et al., 2017). Oil droplets coated by protein membrane can be more evenly distributed in the gel network of protein, which reduce interference with the protein gel network, the protein membrane interacts with the protein matrix to improve the overall gel strength (Zhang, Xie, et al., 2023). Combined with our above findings, L-Lys can promote the small and uniformly distributed oil droplets are filled in the gel network structure, which plays a good supportive role and further improves the gel strength of the emulsion gel. The filled oil droplets also limited the loss of water within the emulsion gel, reducing the CL. In this study, emulsion gels containing filled oil droplets exhibited better WHC and gelation properties compared to pure protein gels. The higher the stability of the emulsion system, the more conducive it is to constructing a stable emulsion gel structure.

### 3.10 LF- NMR and MRI

**Fig. 8:**
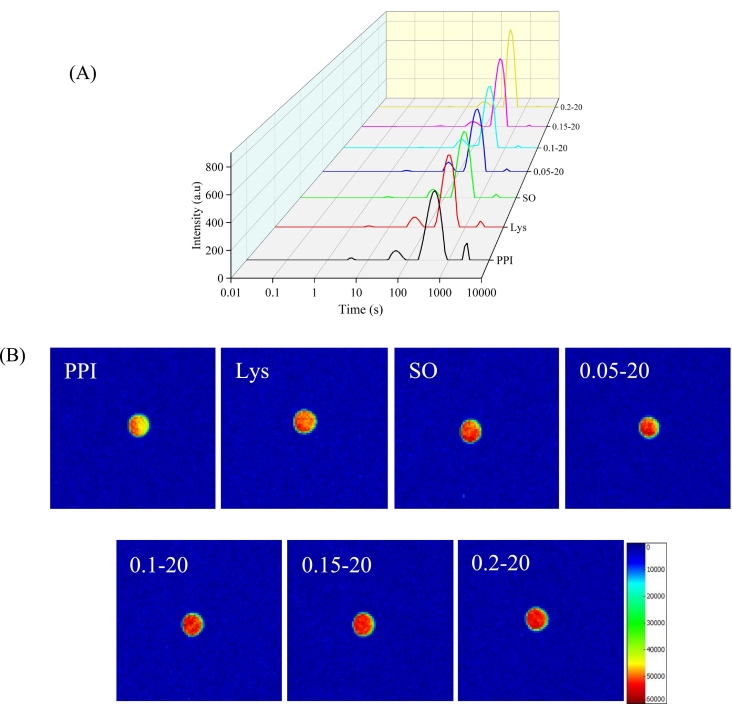
Typical curve of T_2_ relaxation time (A) and MRI images (B) of PPI solution or emulsion gels in different treatment ways.

As shown in Fig. 8A, T_2b_ and T_21_ between 1 and 10 ms and 10–100 ms represent bound water, T_22_ between 100 and 1000 ms represents immobilized water, and T_23_ between 1000 and 10,000 ms represents free water. The longer T_2_ corresponded to the better mobility of water, while the shorter T_2_ the better the ability of the water to bind to the gel matrix (Yang et al., 2020). As shown in Table 2, the relaxation time of bound, immobilized and free water of the gel in the treatment group was reduced compared with that of the PPI group, indicating that water migration was affected by L-Lys and sunflower oil. After adding L-Lys to the emulsion system, the relaxation time was further reduced, suggesting the addition of L-Lys may affect the forces acting between tightly bound water molecules and proteins in the emulsion gels. The changes in water distribution may be related to protein denaturation and aggregation (Li et al., 2019). Which means that the addition of L-Lys to PPI emulsion gels may lead to changes in the protein structure, with water becoming trapped within it.

**Table 2:** T_2_ relaxation time and PT_2_ peak ratio of PPI emulsion gels in different treatment ways.

Measurement indicators	Sample						
	PPI	Lys	SO	0.05–20	0.1–20	0.15–20	0.2–20
T_2b_ (ms)	4.07 ± 0.03^a^	3.59 ± 0.09^b^	2.87 ± 0.06^d^	3.17 ± 0.11^c^	2.71 ± 0.05^de^	3.65 ± 0.08^b^	2.57 ± 0.08^e^
T_21_ (ms)	51.11 ± 1.30^a^	48.65 ± 1.57^ab^	47.58 ± 1.94^ab^	46.59 ± 1.25^bc^	42.86 ± 1.33^cd^	41.41 ± 1.68^de^	38.00 ± 1.56^e^
T_22_ (ms)	456.28 ± 9.14^a^	397.06 ± 12.76^b^	384.53 ± 16.58^bc^	378.12 ± 16.12^bc^	355.57 ± 13.50^c^	299.94 ± 8.15^d^	295.68 ± 12.29^d^
T_23_ (ms)	2826.37 ± 31.30^a^	2450.14 ± 27.78^b^	2459.37 ± 40.65^b^	2444.01 ± 52.44^b^	2434.58 ± 34.48^b^	2412.97 ± 27.66^b^	2374.35 ± 66.86^b^
P_2b_ (%)	0.89 ± 0.03^a^	0.78 ± 0.03^b^	0.26 ± 0.04^de^	0.29 ± 0.04^d^	0.20 ± 0.02^e^	0.52 ± 0.03^c^	0.33 ± 0.03^d^
P_21_ (%)	7.95 ± 0.04^a^	7.47 ± 0.04^b^	6.64 ± 0.09^c^	6.36 ± 0.03^d^	6.73 ± 0.03^c^	6.39 ± 0.05^d^	6.29 ± 0.04^d^
P_22_ (%)	82.95 ± 0.06^e^	84.23 ± 0.42^e^	87.72 ± 0.72^d^	89.13 ± 0.80^cd^	89.51 ± 0.44^bc^	90.80 ± 0.47^b^	92.68 ± 0.63^a^
P_23_ (%)	8.22 ± 0.04^a^	7.72 ± 0.05^b^	5.38 ± 0.03^c^	4.09 ± 0.08^d^	3.48 ± 0.10^e^	2.12 ± 0.10^f^	1.303 ± 0.10^g^

Note: Different letters on the same line (a–g) indicate significant differences between different treatment groups under the same indicator (*P* < 0.05).

The P_2b_, P_21_, P_22_ and P_23_ represent the proportions of different components of water in the gels. As shown in Table 2, immobile water accounted (P_22_) for the largest proportion (>80 %), followed by bound water (P_2b_ and P_21_) and free water (P_23_), indicating that the vast majority of water in the gels was not in the bound state, which also demonstrated that the bound water was not the primary factor in WHC differences. With the addition of L-Lys, emulsion gels showed an increase in immobilized water (P_22_) content and a decrease in free water (P_23_) content, the mobility of water decreased, and the distribution of the water state in the emulsion gel matrix was also dose-dependent with respect to L-Lys. Such a result was also consistent with the CL. The dense gel network as well as the filled oil droplets can also trap water molecules (Cai et al., 2022). Which was also confirmed in this experiment. The addition of L-Lys makes the PPI emulsion gel network structure denser and the pores are reduced, resulting in the retention of water in the gel network structure and the weakening of water mobility. And the uniform size of the oil droplets filled in the network structure, which reduces the water flow channels and further reduces the loss of water.

The gels after different treatments showed significant differences in moisture distribution, which can be visualized by MRI. The low signal intensity (dark or blue areas) indicated relatively free water, while high signal intensity (bright or red) reflected bound or immobile water associated with macromolecules, the more red distribution, the more moisture content of the gel (Li, Fan, et al., 2022). As shown in Fig. 8B, the red region of the PPI gel increased after the addition of L-Lys and sunflower oil. After the addition of L-Lys to the emulsion system, the red region in the PPI emulsion gel was further enlarged and distributed more uniformly with the increasing addition of L-Lys, indicating the immobilized or bound water content increased. These results confirmed that the addition of L-Lys and sunflower oil was effective in retaining water in the gel network and that L-Lys was effective in improving the water retention of PPI emulsion gels. This is consistent with these results obtained by CL and LF-NMR.

### 3.11 FTIR

**Fig. 9:**
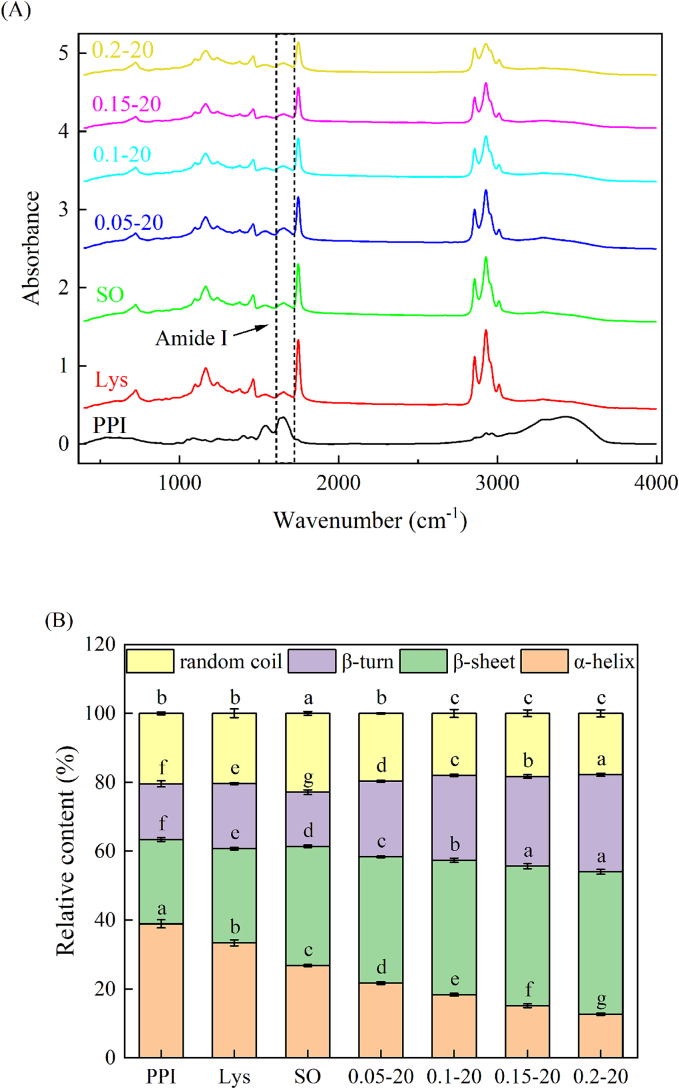
FTIR spectrum (A) and relative content of secondary structure (B) of PPI solution or emulsion gels in different treatment ways.

During thermal induction, proteins undergo partial denaturation and irreversible aggregation to form a three-dimensional gel network structure, making it necessary to study the structural changes in the gels (Hu et al., 2023). As shown in Fig. 9A, distinct polar bonds and functional groups within proteins display unique bands in their infrared spectra (He et al., 2024). Research has indicated that the absorption peaks in the range of 1600–1700 cm^−1^ belonging to the amide I band play a significant role in determining the secondary structure of proteins, representing the stretching vibrations of the C=O bond (Li, Dai, et al., 2023). The relative content of the calculated secondary structure is shown in Fig. 9B. The addition of L-Lys and sunflower oil made PPI gel have lower *α*-helix content and higher *β*-sheet content. Compared with the SO group, after adding L-Lys to the emulsion gel system, there was a dose-dependent the *α*-helix content of PPI emulsion gel decreased (decreased by 52.65 %) and the *β*-sheet and *β*-turn increased (increased by 19.53 % and 78.97 % respectively) in the amount of L-Lys added, the *α*-helix content was the lowest and the *β*-sheet and *β*-turn content was the highest at the addition of 0.2 % L-Lys (*P* < 0.05). The rearrangement and clustering of protein molecules effected by L-Lys might potentially result in this alteration, which is known to bind to the charged residues of proteins through electrostatic interaction, affecting the stability of the protein secondary structure (Zhu et al., 2024). L-Lys disrupts the hydrogen bonds that stabilize the *α*-helix, increasing the flexibility of the protein's structure and dissociating and tending to unfold the protein conformation (Hu et al., 2015). Xu, Guo, et al. (2024) reported that L-Lys leads to unfolding of the MP *α*-helix structure. This is consistent with the results of the present study. Correlation studies have shown that the content of *α*-helix is negatively correlated with gel strength (Zhang, Meng, et al., 2023), and well-structured protein gels typically exhibit an increased abundance of *β*-configurations and a decreased proportion of random coils (Xu, Liang, et al., 2024). Which was probably the main reason for the enhancement of the elastic of emulsion gels. These results confirm that the addition of L-Lys can change the secondary structure of PPI emulsion gels, promote the transition of PPI emulsion gel conformation from *α*-helix to *β*-sheet and *β*-turn, facilitate the strong binding between water-protein and protein-oil droplets, and reduce the water mobility, thus improving the gel strength and WHC of PPI emulsion gels.

## 4 Conclusions

This study indicated that the addition of L-Lys under neutral conditions could enhance the stability of PPI-sunflower oil emulsion. And improve the texture, water retention, rheological properties, and microstructure of PPI-sunflower oil emulsion gels. L-Lys promoted the adsorption of PPI at the O/W interface, leading to the formation of smaller and more uniformly distributed emulsion droplets. Which promoted the interaction and cross-linking between protein molecules during the heat-induced process, and contributed to the formation of highly elastic, structurally homogeneous and dense PPI emulsion gels and reduced water loss. With the increase of L-Lys addition, the PPI emulsion gel properties were enhanced, especially the best PPI emulsion gel properties were obtained with the addition of 0.2 % L-Lys. In addition, a positive correlation between emulsion stability and gel properties of emulsion gel was confirmed. These findings can help to improve the processing utilization of PSE-like chicken meat, and provide new horizons for further development of PSE-like chicken meat protein-based emulsion gel nutrient delivery systems and 3D printed food products. However, this study only focuses on the protein mimetic system, while its potential and effect in practical applications still need to be further explored and verified.

## CRediT authorship contribution statement

**Lin-Meng Wang:** Writing – review & editing, Writing – original draft, Visualization, Investigation, Formal analysis, Data curation. **Yi-Xue Zhang:** Writing – original draft, Visualization, Investigation, Formal analysis, Data curation. **Qing Yang:** Writing – original draft, Investigation, Data curation. **Ke Li:** Writing – review & editing, Visualization, Resources, Methodology, Funding acquisition, Formal analysis. **Wu-Chao Ma:** Writing – original draft, Visualization, Conceptualization. **Yan-Hong Bai:** Supervision, Conceptualization.

## Declaration of competing interest

The authors declare that they have no known competing financial interests or personal relationships that could have appeared to influence the work reported in this paper.

## Data Availability

Data will be made available on request.

## Appendix A Supplementary data

Supplementary material: Schematic diagram of PPI extraction and PPI emulsion and emulsion gel preparation. Image 1
