# Supplementary material for: A comparative study on the PSE-like chicken breast meat protein isolate-sunflower oil emulsion stability, rheological properties and gel properties by different additions of l-Lysine
Source: Food Chem X. 2025 Jun 21;29:102684. doi: 10.1016/j.fochx.2025.102684 (PMC12268901; doi:10.1016/j.fochx.2025.102684)
Supplement: Supplementary file 1 — Supplementary material: Schematic diagram of PPI extraction and PPI emulsion and emulsion gel preparation. [file mmc1.docx]

**Supplementary I**


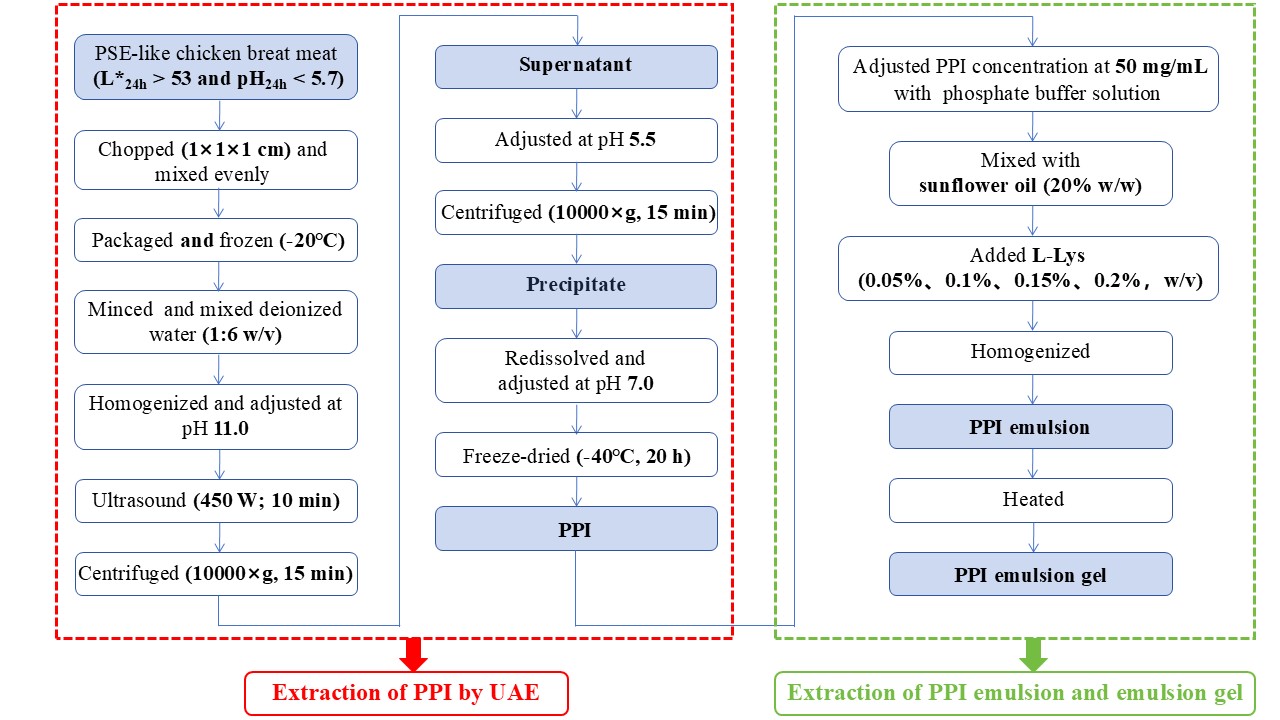


Fig. S1 Schematic diagram of PPI extraction and PPI emulsion and emulsion gel preparation
